# Implementing Eccentric Resistance Training—Part 1: A Brief Review of Existing Methods

**DOI:** 10.3390/jfmk4020038

**Published:** 2019-06-24

**Authors:** Timothy J. Suchomel, John P. Wagle, Jamie Douglas, Christopher B. Taber, Mellissa Harden, G. Gregory Haff, Michael H. Stone

**Affiliations:** 1 Department of Human Movement Sciences, Carroll University, Waukesha, WI 53186, USA; 2 Directorate of Sport, Exercise, and Physiotherapy, University of Salford, Salford, Greater Manchester M6 6PU, UK; 3 Kansas City Royals, Kansas City, MO 64129, USA; 4 High Performance Sport New Zealand, Mairangi Bay, Auckland 0632, New Zealand; 5 Department of Physical Therapy and Human Movement Science, Sacred Heart University, Fairfield, CT 06825, USA; 6 Department of Sport, Exercise, and Rehabilitation, Northumbria University, Newcastle-Upon-Tyne M66PU, UK; 7 Centre for Exercise and Sports Science Research, Edith Cowan University, Joondalup WA 6027, Australia; 8 Center of Excellence for Sport Science and Coach Education, East Tennessee State University, Johnson City, TN 37614, USA

**Keywords:** tempo training, flywheel overload training, accentuated eccentric loading, plyometric training

## Abstract

The purpose of this review was to provide a physiological rationale for the use of eccentric resistance training and to provide an overview of the most commonly prescribed eccentric training methods. Based on the existing literature, there is a strong physiological rationale for the incorporation of eccentric training into a training program for an individual seeking to maximize muscle size, strength, and power. Specific adaptations may include an increase in muscle cross-sectional area, force output, and fiber shortening velocities, all of which have the potential to benefit power production characteristics. Tempo eccentric training, flywheel inertial training, accentuated eccentric loading, and plyometric training are commonly implemented in applied contexts. These methods tend to involve different force absorption characteristics and thus, overload the muscle or musculotendinous unit in different ways during lengthening actions. For this reason, they may produce different magnitudes of improvement in hypertrophy, strength, and power. The constraints to which they are implemented can have a marked effect on the characteristics of force absorption and therefore, could affect the nature of the adaptive response. However, the versatility of the constraints when prescribing these methods mean that they can be effectively implemented to induce these adaptations within a variety of populations.

## 1. Introduction

Eccentric (ECC) muscle actions involve the active lengthening of muscle tissue against an external force or load [1], in contrast to isometric (ISO) and concentric (CON) muscle actions which involve no change in muscle length or the shortening of muscle tissue, respectively. It is well documented that skeletal muscle can produce more relative force during ECC muscle actions than ISO or CON actions [2]. This advocates the use of more efficaciously loaded ECC resistance exercise in the training regimes of athletes, whereby exercise prescription is not constrained by CON strength capacity as in traditional resistance training (TRT; training that typically includes ECC and CON muscle actions but is constrained by CON strength) [3]. Furthermore, there are several idiosyncratic characteristics and acute physiological responses observed with ECC actions that contribute to a novel adaptive signal within the neuromuscular system [4]. Muscle-tendon unit (MTU) architectural and morphological responses include the addition of sarcomeres in series, preferential fast twitch muscle fiber hypertrophy, increase in type IIx fiber composition, and development of a stiffer MTU [3]. Collectively, these adaptations are thought to reflect a shift towards a faster, more explosive muscle phenotype [5]. In terms of MTU mechanical qualities, chronic ECC resistance training, in some studies, has induced greater enhancements in strength [6,7], power [8,9], and stretch-shortening cycle (SSC) function (i.e., ECC/CON function) [9] compared with TRT alone. Although studies have tended to compare ECC and TRT approaches, the distinctive nature of the physiological response should be appreciated. Nonetheless, it appears that neuromuscular development can be maximized when using ECC resistance training. Hence, the integration of eccentrically-emphasized resistance training within an athlete’s physical preparation program appears warranted. The purpose of this review is to provide a physiological rationale for the use of ECC resistance training and to provide an overview of four of the most commonly prescribed ECC training methods.

## 2. Physiological Rationale for Incorporating Eccentric Resistance Training

Supramaximal intensity (>1RM) or heavy load (e.g., ≥85% 1RM) ECC actions have been associated with several novel characteristics, including an augmented anabolic signal, compared with CON or lighter ECC actions [4], and subsequently enhanced neuromuscular changes resulting from chronic ECC resistance training [3]. However, the mechanism behind this phenomenon and several others observed during ECC actions, remain unexplained by traditional beliefs regarding muscle actions (e.g., [10,11]). These unique responses include a greater capacity to produce force [12] and a lower metabolic cost per unit of external work [13] versus CON and ISO action types, and residual force enhancement (i.e., an increase in maximal steady state ISO force immediately following muscle lengthening) [14]. Contemporary representations of muscle action that build upon classical models have substantial explanatory promise [15], and it appears that the structural protein, titin, plays an important contractile role alongside actin and myosin during ECC actions (e.g., increase filament stiffness and force with active muscle stretch) [16]. Concomitant with the molecular differences between muscle action types, the neural strategies of ECC actions also differ in comparison to other muscle action types [17]. Lower surface electromyographic (EMG) activity [18] and motor unit discharge rates [19] have been observed in conjunction with a larger and distinct activation of the motor cortex [20,21]. There is some evidence suggesting a preferential recruitment of high threshold (i.e., fast twitch) motor units with fast ECC actions [22]; however, this has not been a consistent finding [17]. The discrepancy between motor cortex activity and muscle activation reflects a larger voluntary activation deficit observed with maximal ECC versus CON actions [23], and is suggestive of a protective spinal inhibition of ECC force [17], especially in untrained individuals [18,23]. Irrespective of a lower muscle activation, more force (i.e., approximately 20% to 60%) may be produced during maximal ECC versus CON actions across a range of single- and multi-joint movements [24,25], for a lower metabolic demand [26] and less acute fatigue [27]. Furthermore, muscle force production is not constrained by lengthening velocity [23] and therefore very high forces can be achieved with fast muscle lengthening velocities [4]. Logically, there is a markedly higher level of tension generated per active motor unit during maximal or heavy ECC actions, particularly when performed at a high velocity. 

Compared with CON muscle actions, maximal ECC actions have been demonstrated to induce a stronger anabolic signal as evidenced by greater satellite cell activation [28] (i.e., enhanced hypertrophic response), and a greater upregulation of molecular signaling pathways associated with muscle and connective tissue anabolism [29,30]. As a corollary to the tension generated per motor unit during the active stretch of muscle tissue, especially during fast lengthening, there is typically some level of exercise induced muscle damage (EIMD) with the incorporation of ECC actions in training [31,32]. It is not entirely clear if EIMD is a necessary or critical mechanism to promote muscle adaptation [33]; however, it is posited to play a role in subsequent adaptations to ECC training [3]. Irrespective of its role in adaptation, muscle damage remains an important consideration given its association with delayed onset muscle soreness (DOMS) [34] and its acute deleterious effects on measures of neuromuscular performance [35,36]. It is useful to note that the magnitude of EIMD and DOMS resulting from a bout of ECC-only exercise can be attenuated through prior exposure to ECC actions (i.e., the repeated bout effect) [37,38], and therefore can be managed with appropriate planning and dosage of training. Interestingly, satellite cell activation, anabolic signaling, and EIMD following ECC-only actions have been found to be especially pronounced in fast twitch (i.e., type IIa and IIx) muscle fibers [39,40,41], which is suggestive of a fiber type specific response to ECC exercise, potentially stemming from a selective recruitment of these fibers. The adaptive signal induced from eccentrically-emphasized resistance exercise therefore appears to be nuanced and augmented compared with TRT, potentially enhancing subsequent neuromuscular adaptation [4].

A previous meta-analysis showed that when ECC-only exercise was performed at higher intensities compared with CON-only training, total strength and ECC strength increased to a greater extent [42]. Such marked increases in strength with ECC training may be underpinned by an increase in volitional agonist activation (i.e., motor unit recruitment and possibly discharge rates) [17,43], and the downregulation of inhibitory reflexes [44]. ECC-only training appears to be at least as effective as CON-only training in increasing muscle hypertrophy [42,43], which, alongside these neural adaptations, probably contributes to changes in strength. Strength improvements following ECC training tend to be most pronounced when the method of assessment is specific to the type of muscle action mode and velocity used in training [42], although there is evidence for greater improvements in hypertrophy and strength when fast ECC lengthening velocities [45,46] and heavier ECC loads [6] are used. Acknowledging again that due to the ECC force–velocity relationship, these two conditions are not mutually exclusive [4]. While much of the research investigating the effects of ECC training have recruited untrained subjects, improvements in hypertrophy and strength have also been observed in strength-trained participants [7,47]. In addition to increases in strength, improved muscle power output, as assessed under CON-only and SSC (i.e., ECC/CON) conditions, has also been demonstrated [9,48]. Given that power is a product of muscle force and shortening velocity [49], it is likely that increased muscle strength directly contributes to the observed improvements in power output [3]. However, ECC training may be especially effective at increasing power output via novel changes in the underlying architecture or morphology of muscle that reflect a shift towards a more explosive phenotype [5], and a stiffer MTU that is more effective at rapidly transmitting force [50]. 

It has been found that following 10 weeks of ECC-only or CON-only training, muscle hypertrophy was most prominent within the distal part of muscle or mid-portion of the muscle, respectively [51]. This finding is consistent with increases in fascicle length following ECC-only training [52] and ECC/CON training, [53], and is thought to reflect an increase in the number of sarcomeres in series [31]. These adaptations may be due to the stretch-induced strain imposed through muscle during active lengthening [51]. An increase in sarcomeres in series has positive implications for the absolute shortening velocity of the muscle [54], and subsequently, power output [5]. From a morphological perspective, preferential fast twitch fiber hypertrophy following both ECC-only and ECC/CON training [55,56], and possibly an increase in type IIx fiber composition following ECC-only training [46], have been found. It is not entirely clear why these fiber type-specific responses occur, but it is likely related to increased recruitment, tension-generating capacity, and damage of fast twitch fibers with ECC training [3]. Irrespective of the mechanism, an increase in type IIx composition and the relative area of muscle comprised of fast twitch fibers will have a positive impact on muscle fiber shortening velocity [57] and excitation–contraction coupling rates [58], and by extension, power output [5]. Increases in the tendon cross-sectional area [59] and stiffness [60] have also been observed following ECC training, probably due to the absolute load applied to the tissue during heavy ECC actions. An increase in MTU stiffness following ECC training could plausibly attenuate the electromechanical delay (i.e., the interval between muscle activation and the development of external force [2]), and subsequently, aid rate of force development (RFD) and power output [50]. Finally, it is worth noting that the time-course for adaptation is also somewhat characteristic with ECC training, and an extended period (e.g., 6–8 weeks) between the cessation of ECC training and the targeted period for performance peaking (i.e., competition) may be necessary to maximize strength [61] and power [48] outcomes.

In summary, there is a strong physiological rationale for the incorporation of ECC training into the program for an individual seeking to maximize strength and power output. The most substantial benefits tend to be observed following the use of high lengthening velocities and heavy ECC loads. The obvious concern for practical implementation is the risk of EIMD and DOMS and the detrimental effects that these can have on the performance of concurrent training units. However, this can likely be managed with the appropriate periodization of the stimulus. Table 1 summarizes the benefits of ECC training as they relate to hypertrophy, strength, and power.

## 3. Eccentric Training Methods

Strength and conditioning professionals can implement a number of methods to offer a training stimulus throughout the descending (ECC) phase of an exercise. The stimulus usually emphasizes time under tension or mechanical loading, or a combination of both. The current review focuses on tempo ECC training, flywheel inertial training (FIT), accentuated ECC loading (AEL), and plyometric training (PT). The authors are aware that other training methods, such as weightlifting catching and pulling derivatives [62,63,64,65], loaded jumping exercises [66], ECC cycling [67,68], change of direction drills [69], and various sprinting tasks, provide an ECC loading stimulus; however, further details on these methods were not included within this review due to either insufficient evidence or the secondary nature of this training stimulus. Table 2 displays the theoretical hypertrophy, strength, and power training potential of each of the below training methods.

### 3.1. Tempo Eccentric Training

There are many training variables that can be implemented and varied to drive adaptations in athletes [70]. One such method has been termed tempo training or lifting tempo. This method involves altering the time parameters placed on the descending and/or ascending phase of an exercise in an attempt to elicit a specific response [71]. To achieve the desired adaptation, athletes are instructed to lengthen or shorten the pace of an exercise by changing the cadence in which they complete a phase of the movement [72]. 

For example, athletes have been instructed to lift with a tempo of 2/0/2 or 4/0/2, where the cadence for each muscle action would be as follows: ECC/ISO/CON, respectively [72]. Most of the applications of tempo training with a controlled tempo have been placed on increasing the duration of the descending phase of an exercise in an attempt to overload the ECC muscle action [73]. Due to the nature of emphasizing the ECC muscle action inherent to tempo training, this type of training may be better termed slow submaximal ECC exercise since they may not overload the ECC muscle action with regards to exercise intensity (due to the greater force producing capacity of ECC muscle actions). Instead, this approach offers overload through an increase of the duration of muscle tension throughout the descending phase of the exercise. 

Research has examined many permutations of duration implementation for movement tempo and found conflicting evidence as it relates to acute and chronic hypertrophy, strength, and power output [72,73,74,75]. There have been several postulated performance improvements related to intentionally increasing the ECC tempo in resistance exercises. Specifically, researchers have attempted to alter the time parameters to improve hypertrophy and strength through increased ECC muscle tension via increased muscle activation or by increasing the time under tension [76,77,78]. Evidence has been mixed on the effects of extended durations on these performance metrics with some studies showing positive outcomes [77,78], but most showing no favorable outcomes [72,73,79,80,81,82,83]. 

Tension is one of the major drivers of muscular hypertrophy and changes in muscle architecture [84]. In theory, if the duration of tension experienced by the muscle was extended, greater hypertrophy adaptations should be realized [74]. To the authors’ knowledge, only one study showed greater hypertrophy following slow ECC muscle actions compared with fast ECC actions [77]. Muscle thickness, a muscle property related to hypertrophy [85], of the vastus lateralis increased to a greater extent following slow ECC squat training (~4 s) compared with fast ECC squat training (<1 s) in another study [86]. In contrast, a meta-analysis by Schoenfeld et al. [74] indicated that longer ECC actions (>5 s) failed to demonstrate greater hypertrophy compared with traditional training. Beyond muscle hypertrophy and thickness, Stasinaki et al. [86] indicated that fast ECC squat training (<1 s) twice per week for six weeks increased fascicle length by about 10% in novice participants while no notable change followed slow ECC squat training (~4 s). The literature examining the effects of slow or fast tempo ECC training on muscle hypertrophy and architecture is limited. There appears to be mixed findings regarding the benefits of increasing ECC repetition duration when it comes to hypertrophy benefits, while fascicle length increases may favor fast ECC exercise. It is recommended that further research examine these adaptations following both slow and fast tempo ECC exercise in order to explicate its efficacy and place in the resistance training process. 

Another postulated improvement related to increasing the ECC duration of a resistance exercise is the increase in muscle tension resulting in larger strength improvements. Specifically, by extending the duration of the ECC action, an attempt is made to increase the muscle activation and alter fiber recruitment, often measured by surface electromyography (EMG) [78]. Indeed, some studies have demonstrated increased muscle activation with slower ECC actions (e.g., 4–6 s) [76,78], while others have shown the opposite, favoring faster ECC actions (<3 s) or no difference [75,79,83]. When looking beyond EMG studies to dynamic task specific outcomes, the literature has shown that intentionally increasing the ECC duration may result in suboptimal strength adaptations when compared with faster ECC actions. To the authors’ knowledge, only one study could demonstrate greater strength outcomes using a slower tempo compared with a faster tempo in the elbow flexors [77]. In contrast, an abundance of studies support the use of faster ECC actions when attempting to develop and enhance muscular strength, power, and RFD [72,73,79,80,81,82,83,86]. 

#### Applications and Issues 

**Figure 1:**
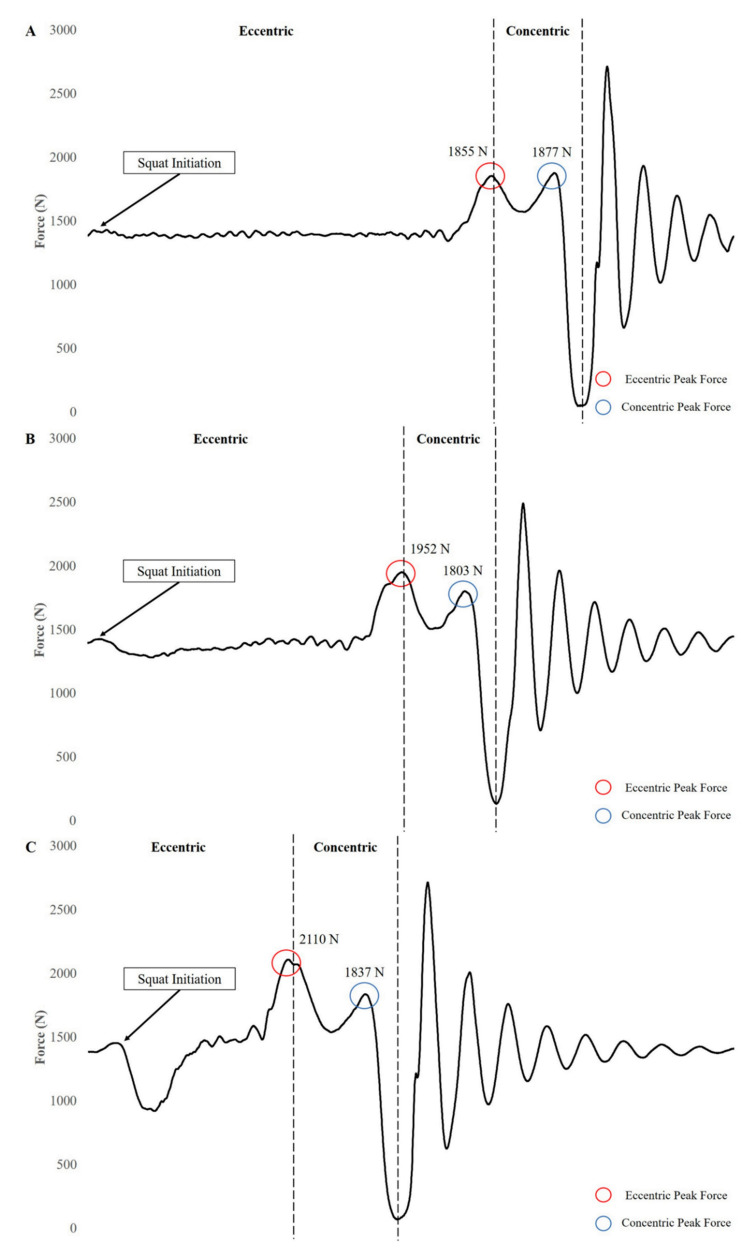
Force-time curves of a four-second tempo (**A**), two-second tempo (**B**), and traditional (**C**) back squat performed with 70 kg by a healthy, 31 year old male.

Several considerations should be taken into account before integrating tempo ECC training into a resistance training program. First, by extending the duration of the ECC muscle action, the absolute load on any given exercise must be lowered for the subject to complete all of the prescribed repetitions [72]. Absolute loading is one factor in the development of strength and may be one of the reasons strength adaptations have not favored slow tempo ECCs. It should also be noted that a longer (controlled) ECC duration may actually limit the magnitude of ECC force production, potentially limiting a strength stimulus (Figure 1). Second, the total amount of volume that can be completed when comparing slow tempo ECCs to fast or self-paced tempos is lower [82,83]. Volume has been identified as one of the drivers of hypertrophy, and therefore if less volume is completed, hypertrophy may be compromised compared with faster or self-selected ECC tempo strategies. However, it should be noted that if volume is determined as the overall time under tension, slow tempo ECC actions may provide a greater stimulus [87]. Third, intentionally slow training may not carry over to sport where fast ECC and SSC actions occur [73]. Finally, increasing ECC duration may result in higher ratings of perceived exertion and greater lactate accumulation compared with faster ECCs, which may result in more discomfort and acute fatigue, comparable or less hypertrophy and strength, and comparable or greater hormonal responses compared with fast ECCs [72,78,88,89,90].

### 3.2. Flywheel Inertial Training

A frequently investigated topic within the ECC training literature involves the use of a FIT device. This type of training was first investigated over 20 years ago as a gravity-independent training method to counteract the deleterious effects of microgravity of skeletal muscle [91]. Flywheel devices use inertial resistance that results from the unwinding of the flywheel’s strap caused by a CON muscle action, which is then followed by a rewinding of the flywheel’s strap, resulting in an ECC muscle action. Simply put, the rate at which the strap is re-wound is based on the rate at which it is unwound, offering resistance through the ECC phase of the exercise. In more mechanistic terms, the resistance imparted on the athlete by the device is dependent on the mass, radius, and angular acceleration of the flywheel. Previous research has indicated that FIT has improved muscle mass [92], maximal voluntary contraction [53,92,93], maximal strength (i.e., 1RM) [94,95], ECC force production [91,96], power output [95,97], jump ability [95,97,98], running velocity [95,98,99], and EMG activity [53,100,101] in both untrained participants and a variety of athletic populations. However, it should be noted that limited research has compared the effects of FIT to TRT and thus, it is difficult to draw concrete conclusions regarding the effectiveness of this method. Additional benefits of using a flywheel device is its versatility, including the ability to perform a variety of exercises in different movement planes [102], as well as the ability to use the device in multiple training locations (i.e., portability).

Several meta-analyses have examined the effectiveness of FIT on muscle hypertrophy, strength, power, and other performance characteristics [103,104,105]. Two of the previous meta-analyses support the use of FIT over free weights and weight stack training [103,104]; however, another meta-analysis disputes these findings and notes that FIT did not provide any additional benefits to muscle strength compared with gravity-dependent resistance training [105]. It should be noted that the latter research group indicated that the previous meta-analysis by Maroto-Izquierdo and colleagues [103] had several methodological shortcomings (e.g., the omission of a traditional training group in 67% of the included studies and the inclusion of both randomized and non-randomized controlled trials) that may question the conclusions drawn [106]. Thus, conclusions regarding the training adaptations following FIT compared with traditional methods of training (e.g., free weights and weight stack training) still require further investigation.

**Figure 2:**
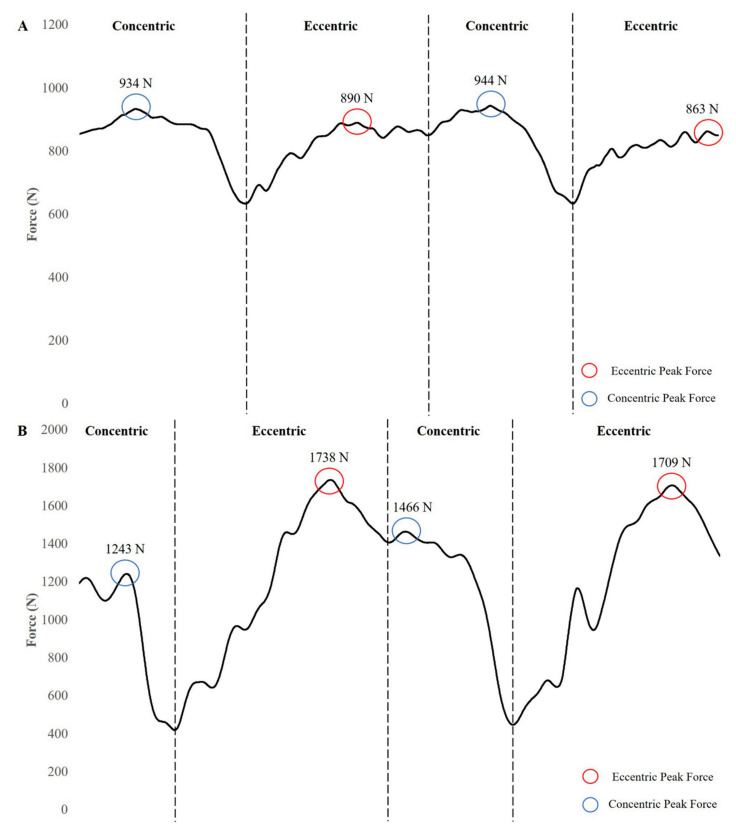
Force–time curves of flywheel squats performed by a healthy, 31 year old male using a 0.050 kg·m^2^ inertial load with a slow (**A**) (~ 3 s) and fast (**B**) (< 1 s) concentric action.

**Figure 3:**
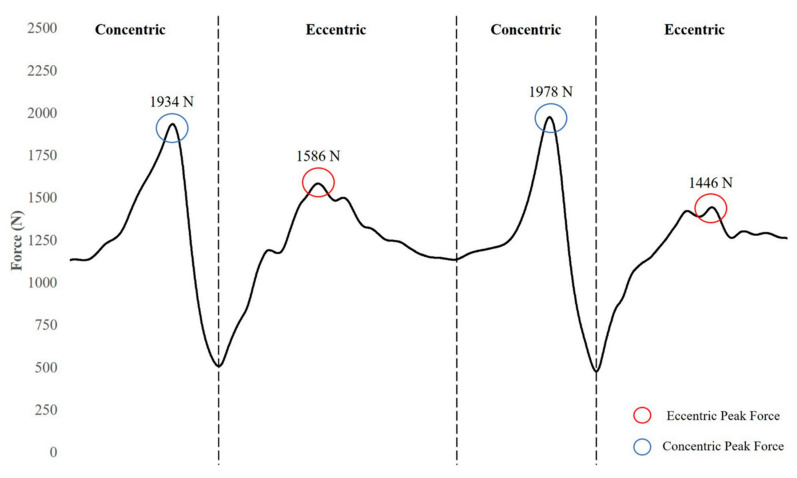
Force–time curve of flywheel squats performed by a healthy, 31 year old male using a 0.050 kg·m^2^ inertial load with a fast concentric action (<1 s) and a slow eccentric action (~2 s).

As discussed previously, a number of studies have shown positive improvements in strength–power characteristics when using flywheel devices within their training. Many researchers have attributed these findings to an “ECC overload” stimulus provided by the flywheel device. However, the law of conservation of energy indicates that energy cannot be created or destroyed in an isolated system. Carroll et al. [107] indicated that a progressive overload stimulus (i.e., greater ECC stimulus) can be provided using a flywheel device; however, the “intensity” of the ECC phase of the movement when using a flywheel device may be altered by the CON velocity generated by the individual. Figure 2 displays the differences in flywheel squat force–time curves using slow and fast CON muscle actions. Thus, some may question if an ECC overload stimulus occurs while using a flywheel device instead of an ECC stimulus equivalent to the energy produced during the CON phase. Previous research suggested that the technique that most effectively applies an ECC stimulus involves resisting the inertial force gently during the first third of the ECC action and subsequently applying a maximal effort to decelerate the rotating flywheel and stop the movement at the end of the range of motion [108]. It should be noted that this technique may not always be adopted by participants. Previous research indicated that increases in wheel size (i.e., inertia) resulted in an increase in ECC peak and mean force in both moderately active men and women; however, there was no additional increase in the ECC stimulus beyond a 0.0375 kg∙m^2^ inertial wheel [109]. These results may be partially explained by the technique that the individuals used to decelerate the larger wheels. For example, the previous study indicated that as inertia increased, ECC duration increased to a small to large extent with men and moderate extent with women. It is possible that the individuals attempted to control the velocity of the squat descent in anticipation of a more challenging stimulus at the end of the movement. It should be noted that performing flywheel repetitions in this manner may reduce the ECC force stimulus experienced by an individual (Figure 3). This appears to be further evidence that the technique adapted by participants may dramatically alter the training stimulus.

Something to consider when interpreting the results of the existing FIT studies is the training status of the individuals being tested. It appears that males and females respond similarly (e.g., muscle mass, strength, and power output) to FIT [94,109]. Interestingly, Sanchez and de Villarreal [104] displayed that only one study included within their meta-analysis included trained participants, while the remaining participants were classified as either sedentary or physically active. However, it should be noted that the original article did not provide any training status information beyond the fact that the participants included first Spanish soccer division club academy players [98]. Moreover, and in contrast to the previous meta-analysis [104], the authors noted that none of the participants had previously used flywheel devices. Another consideration specific to training status is the relative strength levels of the individuals. While several studies provide some measure of the participants’ maximal absolute strength [94,95,97,110,111], a paucity of research provides relative strength measurements [112]. It should be noted that stronger individuals may require greater CON velocities or use larger inertial loads while using flywheel devise to experience an ECC overload stimulus. In contrast, weaker individuals may experience an ECC overload stimulus using a variety of inertial loads due to a limited capacity to tolerate and produce large ECC forces and rates of force production. Finally, it should be noted that previous experience with flywheel devices may modify the training stimulus that an individual may experience [104]. From a practical standpoint, if an individual is accustomed to the transition between the CON and ECC phases of a flywheel, they may be less likely to experience an overload stimulus. For example, if an individual is comfortable descending rapidly into a flywheel squat, a smaller overload stimulus may be provided due to their ability to decelerate their body mass and inertial load rapidly. This appears to be similar in both males and females [109]. Collectively, the literature suggests that FIT may be effectively implemented with those with less training experience and lower strength levels; however, there is a lack of evidence supporting its use with stronger individuals. Finally, like other training methods, an individualized approach should be taken when prescribing this type of exercise. 

When interpreting the findings of the comparative FIT literature, it is important to consider the quality of the prescribed training programs. For example, a number of existing training studies compared training with the flywheel device with weight stack machine training [93,95,110,113] while other studies compared FIT to a control condition that included no additional training [98,111]. The previous studies comparing flywheel and weight stack machine training indicated that greater results were produced with the flywheel device; however, it should be noted that the majority of the training studies used a training program focused on single joint exercises [93,110,113]. Moroto-Izquierdo et al. [95] examined the differences between flywheel and weight stack machine leg presses (control condition). While the flywheel device produced the greater strength–power adaptations (e.g., maximal dynamic strength, power output, jump height, 20 m sprint time, and t-test time), it should be noted that the control program included 4 sets of 7 repetitions at a 7RM. The quality of the control condition training program may be questioned in this instance because the prescribed volume–load may lead to a considerable amount of fatigue, which may mitigate the magnitude of strength–power adaptations. Therefore, it is suggested that practitioners interpret these results with caution. Interestingly, a similar high volume training program was prescribed with a multi-joint exercise (e.g., half-squat) and compared with FIT at the same volume and found contrasting results compared with the previous study [114]. de Hoyo and colleagues [114] indicated that the traditional training group improved their countermovement jump height and 20 m sprint time to a greater extent than the flywheel training group. Collectively, it is clear that further comparative research including higher quality programming within traditional training programs is needed before conclusions regarding the effectiveness of FIT can be made. 

#### Additional Considerations

The ability to effectively prescribe flywheel training for specific fitness goals is limited. While more information has become available in recent years, it is difficult to monitor and adjust training volumes and intensities to fit the needs of the athletes. At present, many of the existing training articles appear to arbitrarily choose sets and repetitions with a given inertial load. For example, a previous systematic review and meta-analysis indicated that many of the studies completed within the past 20 years employed a workload of 4 sets of 7 repetitions over 5 to 15 weeks of training [103]. However, this often leads to “wasted repetitions” (i.e., repetitions needed to get the flywheel up to the desired velocity), training to failure, and poor training load management. Previous research has suggested that lower inertial training loads may produce greater adaptations for peak power, while moderate to high inertial loads may provide greater ECC overload [112,115]. However, as previously noted, larger inertial loads may increase the length of the ECC phase of the movement, which may modify the ECC overload stimulus [109]. Moreover, the familiarity and strength levels of the individuals may alter how the CON and ECC phases of the movement are performed, which again, may alter the ECC overload stimulus experienced. Carroll et al. [107] indicated that velocity may be used to prescribe different intensities when using flywheel devices; however, further research is needed to provide effective training recommendations. 

### 3.3. Accentuated Eccentric Loading

**Figure 4:**
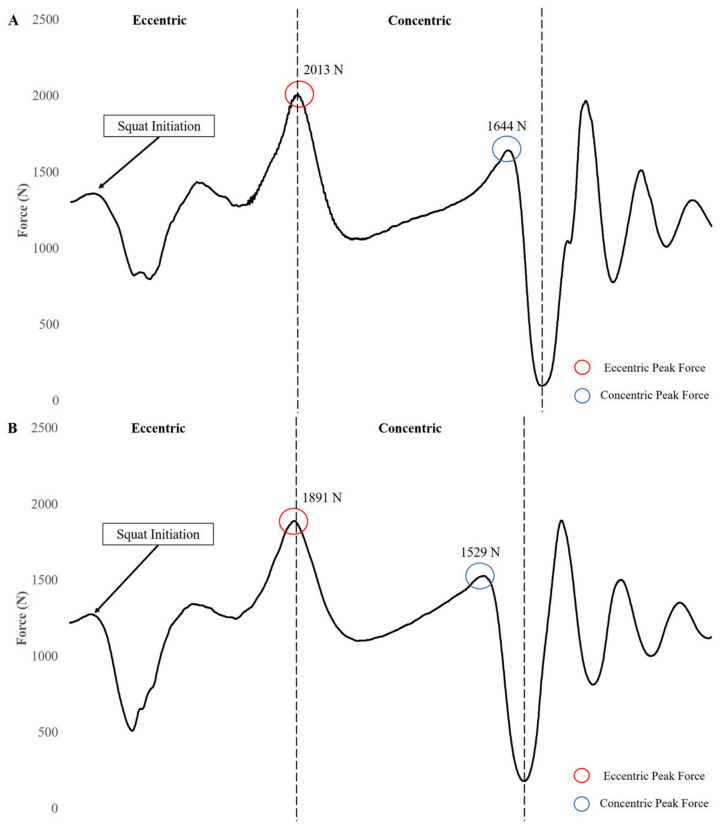
Force–time curves of an accentuated eccentric loaded (**A**) (65 kg + 20 kg on weight releasers) and traditional (**B**) (65 kg) back squat performed by a healthy, 29 year old female.

AEL is another popular application of phase-specific overload—though the term is commonly used to encompass all types of ECC overload training. However, AEL refers to a specific programming tactic in which the ECC load is in excess of the CON load using movements that require coupled ECC and CON actions while providing minimal interruptions to the natural mechanics of the chosen exercise [116]. For example, weight releasers may be used during a squat or bench press to provide greater absolute loading exclusively during the ECC phase. Due to the nature of the devices, the weight is unloaded in the transition from ECC to CON phase seamlessly, causing minimal alteration to the athlete’s technique. Figure 4 displays the differences in force–time characteristics between an AEL back squat using weight releasers and a traditional back squat.

AEL is an advanced training tactic that aims to exploit the established benefits of ECC overload training, like the manipulation of movement cadence or flywheel resistance. However, the higher absolute loading required of AEL makes it an attractive strategy for applying additional stress to the muscle and connective tissue while maintaining the CON stimulus. The ability to preserve mechanical similarity and coupled actions may also explain previous reports of favorable changes in jumping and throwing actions, demonstrating that the training effects from AEL may transfer well to sport tasks and performance when applied to both strength and PT exercises [117,118,119,120,121,122,123,124]. Maintaining technical quality has important practical implications as well as including a reduced risk of injury in its application. 

The chronic application of AEL has also been explored, but a relative paucity of literature currently exists. Due to the greater mechanical tension [125] and work [126,127] demands of AEL during the ECC phase, it may be logically used in an effort to induce muscle hypertrophy. Skeletal muscle response tends to be proportional to the magnitude of mechanical loading, though changes in whole muscle size appear to be similar when comparing traditional loading to AEL. Four studies have examined changes in anatomical cross-sectional area (aCSA) following AEL—three of which observed no difference between AEL and traditional loading [7,55,128]. Further, of the studies that observed no between-group differences in muscle size changes, AEL did produce statistically greater improvements in strength [7,128] and jump performance [55]—likely attributed to changes to the nervous system. Walker and associates [7] observed favorable changes in voluntary muscle activation following AEL compared with traditional loading, supporting this hypothesis. Though not directly observed by Walker and colleagues [7], changes to muscle architecture may partially explain some of the observed increases in strength and power production outcomes following AEL. As previously discussed, ECC muscle actions may elongate muscle fascicles, allowing the muscle to produce higher shortening velocities. Furthermore, because AEL permits minimal interruption to the CON phase, and would therefore not be a detriment to the benefits of traditional loading, it is reasonable that the increases in sarcomeres in-series are also possible with this tactic. Taken together, it is reasonable to infer that AEL may favorably influence muscle structure and strength–power potential. Though logical, such a conclusion can be conjecture at best given the current state of the evidence. Despite the lack of evidence supporting AEL as a favorable tactic in altering muscle size or architecture compared with traditional loading, enhanced anabolic signaling and hormonal response have been observed [55,129,130]. The augmented anabolic environment may explain some of the shifts towards faster myosin heavy chain isoforms and fast-fiber specific cross-sectional area changes using AEL [55,129]. Specifically, Friedmann and associates [129] observed a 320% increase in type IIx mRNA following AEL knee extensions (3 sets of 25 repetitions three times per week for four weeks with 70% and 30% 1RM during the ECC and CON actions, respectively) compared with a 24% decrease following traditional knee extensions (6 sets of 25 repetitions three times per week for four weeks with 30% 1RM for both the ECC and CON actions). Though this outcome did not reach statistical significance, it demonstrates AEL’s efficacy as a resistance training means to increase strength–power potential. In a later study by the same group, statistical increases in type IIx fiber CSA were observed following chronic exposure to AEL knee extensions (5 sets of 8 repetitions three times per week for six weeks with about 1.9 x the 8RM load and 8RM during the ECC and CON actions, respectively) but not traditional knee extensions (6 sets of 8 repetitions three times per week for six weeks with 8RM for both the ECC and CON actions), which were accompanied by significant correlations between these changes and changes in strength [55].

Practitioners may also aim to exploit the mechanistic advantages of AEL to augment the subsequent CON action acutely. The higher absolute ECC loading may theoretically increase the active state of the muscle [131], the Ca^+2^ sensitivity [132], and ECC RFD (RFD_ECC_) [126,127]. All of the aforementioned can likely be uniquely exploited using AEL compared with other ECC training strategies due to the maintenance of tight coupling of the ECC and CON action, thus making it a rational acute potentiation strategy. 

Despite supporting evidence and sound theoretical basis, the efficacy of AEL requires thoughtful consideration due to the inconsistency in the methodological details of the existing evidence. When applying AEL to acutely enhance CON performance (e.g., improved power output, RFD, etc.), the inherent balancing act between fatigue and potentiation must also be considered [133,134]. Contractile history can have both fatiguing and potentiating effects on skeletal muscle performance [135] and such physiological sensitivity may explain the mixed results observed thus far using AEL to acutely augment force production characteristics. For example, Doan and associates [136] observed enhanced 1RM performance prescribing supramaximal loads using the bench press. Despite using similar absolute intensities and exercise selection, the findings of Ojasto and Häkkinen [137] disagreed with those of Doan and associates [136]. The latter group reported that subsequent 1RM and CON force production both significantly diminished using AEL. Because of the mixed results, some have explored the kinetic and kinematic characteristics of AEL within the context of resistance training [126,127]. The most notable finding is the increased RFD_ECC_ using AEL compared with traditional loading [126,127], which provides support of the efficacy of this training tactic in satisfying the mechanistic criteria to induce CON potentiation. When ECC muscle actions are rapid and forceful (i.e., high RFD_ECC_), then it is possible that greater muscle spindle activation [138] or stretch to the MTU occurs [139]. Therefore, it would be expected that enhancement of the subsequent CON performance would occur. However, this was not observed by Wagle and associates [126,127], citing the individualized nature of potentiation [137] or a suboptimal CON:ECC loading ratio as potential rationale. 

Conservative loading is more typical of programming decisions using plyometric exercise selection compared with resistance training. Further, the nature of the technique of plyometrics may create a more favorable situation for the athlete to return stored energy provided via AEL to potentiate explosive performance. Even so, the outcomes observed in applying AEL to plyometrics are similarly inconsistent. Moore and associates [140] failed to induce potentiation using a variety of loading parameters in the jump squat, even with conservative CON loading and trained subjects. The findings of this group highlight the potential importance of exercise selection. The large range of motion required in jump squats may have been inappropriate in eliciting favorable explosive performance outcomes, as it is reasonable that an extended amortization phase and subsequently diminished use of the SSC occurred, though not directly observed [131,141]. Sheppard and colleagues [123] may have mitigated some of these potential shortcomings by using countermovement jumps—likely by having a smaller ECC displacement compared with the full jump squats. The observed outcomes were in contrast to those of Moore and associates [140], with AEL inducing statistically greater jump heights, peak power, and peak velocity compared with traditional countermovement jumps [123]. The difference in findings may be due to the previously mentioned influence of exercise selection. Sheppard and colleagues [123] also used a different loading strategy at a much lower intensity, using 20-kg dumbbells that were dropped at the bottom of the descent. This may have permitted a more rapid return of stored energy and enhanced jump performance [131,141]. 

Though still a relatively under-researched ECC training method, AEL has shown enough promise under both acute and chronic conditions to warrant further investigation. Future research should focus on the details of programming decisions, such as the differences in phase-specific intensity—particularly to differentiate between active lowering of a submaximal load and maximal or submaximal ECC prescription (i.e., resisting deformation), as this may be one of the contributory factors for the inconsistency in the findings of the existing literature. Additionally, exercise selection and proper timing within a periodized training plan should be explored. Readers interested in a more thorough summary and interpretation of the existing AEL literature are encouraged to examine Wagle and associates’ review on the topic [116]. 

### 3.4. Plyometric Training 

Another form of resistance training that may provide a unique ECC stimulus is PT. Plyometric exercises may be defined as rapid ballistic movements that incorporate the use of the SSC [142]. Specifically, plyometric exercises use an ECC muscle action (i.e., MTU lengthening) to enhance a subsequent CON muscle action (i.e., MTU shortening) by using stored elastic energy from the MTU created by the active ECC phase of the movement [143,144]. Through the use of these ECC–CON actions, an individual may increase force, power output, and RFD. This in turn may lead to improvements in athletic performance, but may also have a positive effect on injury prevention via improved movement biomechanics, strength, and balance [145]. 

**Figure 5:**
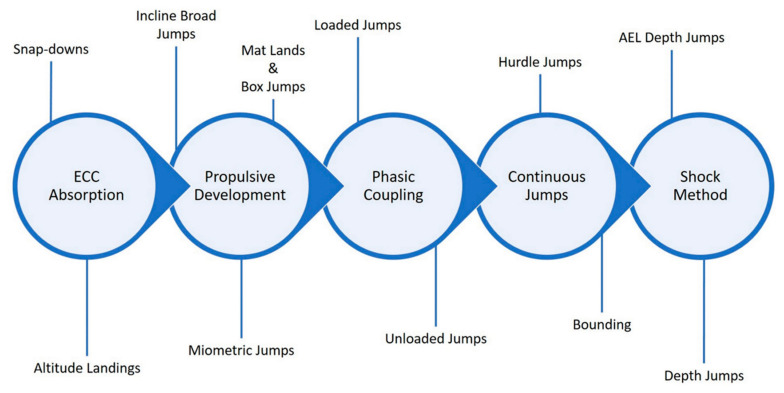
Plyometric training phasic goals and associated exercises.

Unlike the previous training methods, PT may not fall exclusively into the ECC training category. However, it should be noted that each of the previously discussed methods may improve SSC function by enhancing the ECC force production characteristics, which in turn may enhance the CON phase of the movement [3]. While the purpose of using plyometric exercises may be to improve CON phase characteristics, it should be noted that an overload stimulus during the ECC phase of the movement may lead to further adaptations during both the ECC and CON phases of the movement. Specifically, the overload stimulus of specific plyometric exercises may be based on the velocity of the ECC phase or rate of loading [146]. Verkhoshansky [147] discussed miometric (i.e., rapid CON action without a countermovement), ISO–miometric (i.e., rapid CON action without a countermovement using muscles that previously were under ISO tension), plyometric–miometric (i.e., rapid CON action following a countermovement), and “shock” (i.e., rapid CON action following a countermovement that resulted from an involuntary falling impact) variations of ballistic muscle actions. It was also noted that the “shock” method may produce the greatest power outputs due to greater magnitudes of muscle activation. This is supported by more recent research that showed that depth jumps possess greater rates of mechanical loading compared with other exercises [148]. While depth jumps and drop jumps may increase the ECC velocity of the movement due to dropping from a raised surface, it should be noted that other bilateral (e.g., cone/hurdle hops, countermovement jumps, broad jumps, etc.) and unilateral (e.g., bounding, power skipping, skating, etc.) plyometric exercises may also be used to provide an ECC loading stimulus. However, it should be noted that this stimulus may vary based on the nature of the plyometric exercise (Figure 5).

A considerable amount of research has been completed over the past three decades regarding the effectiveness of PT for improved athletic performance. Due to the abundance of PT literature, several meta-analyses have been completed. Such analyses have indicated that lower extremity PT may enhance jump [149,150,151,152,153], sprint [154], and change of direction performance [155,156], as well as muscular strength [157]. Another meta-analysis concluded that the incorporation of PT may help prevent injuries in youth sport athletes [158]. Further research has indicated that upper extremity PT may enhance baseball throwing velocity [159], tennis serve velocity [160], and medicine ball throwing performance [161]. It is important to note that the PT literature has included a wide variety of participants (e.g., males, females, untrained, trained, youth, adolescent, etc.) with different sport backgrounds. For this reason, it is important to put the findings of the extant literature into context to aid in the effective implementation of plyometric exercise for each population. 

While there appears to be substantial support for the use of PT within a holistic training program that aims to improve athletic performance, it should be noted that the way plyometric exercises are implemented may result in different magnitudes of training adaptations. Specifically, the plyometric exercises chosen, the athletic population being trained, the other methods of training that are being used, the season that the athlete is currently in, and the training adaptations sought may drastically modify how plyometric exercises may be implemented. Several studies [162,163,164,165,166,167,168] have sought to identify the intensity of different plyometric exercises by using a variety of ECC (e.g., rate of force development, joint power absorption, joint reaction forces), CON (e.g., peak force, time to takeoff, muscle activation), and landing variables (e.g., impulse, time to stabilization, rate of force development). As displayed in Figure 5, these exercises may fall within an intensity spectrum, which may allow for the intentional prescription of low, moderate, and high intensity exercises. It is important to remember that as the intensity of plyometric exercises increases, the volume of contacts should decrease to prevent excessive fatigue and allow for recovery [169,170]. The current discussion has focused almost exclusively on lower extremity plyometric exercises primarily due to the paucity of literature dealing with upper extremity and rotational plyometric exercises. However, it should be noted that the literature that focuses on the latter methods of training is centered almost exclusively on rehabilitation, which is beyond the scope of this review. 

Another consideration when prescribing plyometric exercises is the training status of the athlete. While plyometric exercises may be prescribed to athletes with different training experience, the exercises selected should be based on the athlete’s ability to tolerate the training stress associated with each method. Previous literature indicated that care needs to be taken with younger athletes to ensure that the PT stress remains low until the athlete develops the capacity to tolerate the exercise [152,153]. Further research indicated that while younger athletes may improve their change of direction performance using plyometric exercise, older athletes may display greater benefits [156]. It should be noted that stronger individuals may receive greater training benefits from PT compared with weaker individuals [171,172]. In summary, it is important to begin a PT progression with youth athletes with low intensity plyometric exercises with an appropriate training volume before progressing them to higher training volumes and eventually higher intensity plyometric exercises.

A final consideration when implementing plyometric exercises in training is the other types of training that may be prescribed concurrently. A meta-analysis indicated that PT may produce similar vertical jump adaptations compared with weightlifting [173]; however, two studies that have compared the two training methods suggested that greater adaptations were produced as a result of training with weightlifting movements [174,175]. It should be noted that greater improvements in performance have been shown when PT is combined with other resistance training methods compared with using PT alone [176,177,178]. In contrast, some studies have shown no difference between methods [179,180,181]. However, it should be noted that the combined training groups in the latter studies had a larger training volume compared with the other groups, which may result in a greater magnitude of fatigue, preventing potential adaptations from occurring within the testing window following training. While improvements can occur when using multiple training methods concurrently, practitioners need to be aware of the training stress associated with the volume of combined training to ensure the desired adaptations occur. 

## 4. Recommendations for Future Research

Although the current review has provided an overview of four of the primary methods of ECC resistance training, there are a number of research questions that require further investigation. Generally speaking, future research should examine how long the residual effects of ECC training last, how often should ECC training be prescribed in different training phases, and how fatigue management strategies differ compared with traditional methods of training. Specific to tempo ECC training, it is important to identify the optimal length of the ECC phase to determine the best practices for the training adaptation that is sought. Furthermore, future research examining tempo training should focus on the chronic effects of submaximal tempo training on hypertrophy, strength, and power as limited research currently exists. Regarding FIT, it is suggested that future research examine performance changes as they relate to motor learning and changes in work capacity, muscle architecture, and relative strength. Furthermore, researchers should consider examining exercise technique changes following FIT. Future research using AEL as a training method should consider examining the optimal length of the ECC phase of AEL protocols during different training blocks to examine the differences in muscle architectural and neuromuscular adaptations. Further research may also consider examining the implementation of AEL using different set configurations to determine the most effective prescription strategies for different athletic populations. While the majority of the PT research supports the prescription of two days per week, future research should examine the effects of undulating intensity days within each week of training compared with using the same intensity both days.

## Figures and Tables

**Table 1:** Summary of underlying eccentric training effects that may benefit hypertrophy, strength, and power output.

Hypertrophy	Strength	Power Output
↑ Anabolic signaling ↑ Satellite cell activation ↑ Motor unit recruitment ↑ Activation of motor cortex ↑ Force production capacityPossible ↑ fast twitch motor unit preferential recruitment	↑ Motor unit recruitment ↑ Activation of motor cortex ↑ Force production ↑ Motor unit discharge rate ↑ MTU stiffness ↓ Regulation of inhibitory reflexesPossible ↑ fast twitch motor unit preferential recruitmentPossible ↑ type IIx fiber composition (phenotype shift)	↑ Motor unit recruitment ↑ Activation of motor cortex ↑ Force production capacity ↑ Motor unit discharge rate ↑ MTU stiffness ↓ Regulation of inhibitory reflexes ↑ Muscle fascicle lengthPossible ↑ fast twitch motor unit preferential recruitmentPossible ↑ type IIx fiber composition (phenotype shift)Possible ↑ excitation–contraction coupling rates ↑ Muscle fiber shortening velocity

**Table 2:** The theoretical potential of eccentric training methods to benefit hypertrophy, strength, and power output.

Eccentric Training Method	Hypertrophy	Strength	Power
Tempo Eccentric Training	++	+	+
Flywheel Inertial Training	+++	++	++
Accentuated Eccentric Loading	+++	+++++	++++
Plyometric Training	+	++	++++

+ = low potential; +++ = moderate potential; +++++ = high potential.

## References

[1] Lindstedt S.L., LaStayo P.C., Reich T.E. (2001). When active muscles lengthen: Properties and consequences of eccentric contractions. News Physiol. Sci..

[2] Prilutsky B.I., Zatsiorsky V.M. (2000). Eccentric muscle action in sport and exercise. Biomechanics in Sport: Volume IX Encyclopaedia of Sports Medicine.

[3] Douglas J., Pearson S., Ross A., McGuigan M.R. (2017). Chronic adaptations to eccentric training: A systematic review. Sports Med..

[4] Douglas J., Pearson S., Ross A., McGuigan M.R. (2017). Eccentric exercise: Physiological characteristics and acute responses. Sports Med..

[5] Vogt M., Hoppeler H.H. (2014). Eccentric exercise: Mechanisms and effects when used as training regime or training adjunct. J. Appl. Physiol..

[6] English K.L., Loehr J.A., Lee S.M.C., Smith S.M. (2014). Early-phase musculoskeletal adaptations to different levels of eccentric resistance after 8 weeks of lower body training. Eur. J. Appl. Physiol..

[7] Walker S., Blazevich A.J., Haff G.G., Tufano J.J., Newton R.U., Häkkinen K. (2016). Greater strength gains after training with accentuated eccentric than traditional isoinertial loads in already strength-trained men. Front. Physiol..

[8] Gross M., Lüthy F., Kroell J., Müller E., Hoppeler H., Vogt M. (2010). Effects of eccentric cycle ergometry in alpine skiers. Int. J. Sports Med..

[9] Elmer S., Hahn S., McAllister P., Leong C., Martin J. (2012). Improvements in multi-joint leg function following chronic eccentric exercise. Scand. J. Med. Sci. Sports.

[10] Huxley A.F., Niedergerke R. (1954). Structural changes in muscle during contraction: Interference microscopy of living muscle fibres. Nature.

[11] Huxley H.E., Hanson J. (1954). Changes in the cross-striations of muscle during contraction and stretch and their structural interpretation. Nature.

[12] Linari M., Lucii L., Reconditi M., Casoni M.E., Amenitsch H., Bernstorff S., Piazzesi G., Lombardi V. (2000). A combined mechanical and x-ray diffraction study of stretch potentiation in single frog muscle fibers. J. Physiol..

[13] Curtin N.A., Davies R.E. (1975). Very high tension with very little atp breakdown by active skeletal muscle. J. Mechanochem. Cell Motil..

[14] Edman K.A.P., Elzinga G., Noble M.I.M. (1982). Residual force enhancement after stretch of contracting frog single muscle fibers. J. Gen. Physiol..

[15] Herzog W., Powers K., Johnston K., Duvall M. (2015). A new paradigm for muscle contraction. Front. Physiol..

[16] Herzog W. (2014). The role of titin in eccentric muscle contraction. J. Exp. Biol..

[17] Duchateau J., Baudry S. (2014). Insights into the neural control of eccentric contractions. J. Appl. Physiol. (1985).

[18] Aagaard P., Simonsen E.B., Andersen J.L., Magnusson S.P., Halkjaer-Kristensen J., Dyhre-Poulsen P. (2000). Neural inhibition during maximal eccentric and concentric quadriceps contraction: Effects of resistance training. J. Appl. Physiol. (1985).

[19] Del Valle A., Thomas C.K. (2005). Firing rates during strong dynamic contractions. Muscle Nerve.

[20] Fang Y., Siemionow V., Sahgal V., Xiong F., Yue G.E. (2004). Distinct brain activation patterns for human maximal voluntary eccentric and concentric muscle actions. Brain Res..

[21] Fang Y., Siemionow V., Sahgal V., Xiong F., Yue G.H. (2001). Greater movement-related cortical potential during human eccentric versus concentric muscle contractions. J. Neurophysiol..

[22] Nardone A., Romano C., Schieppati M. (1989). Selective recruitment of high-threshold human motor units during voluntary isotonic lengthening of active muscles. J. Physiol..

[23] Amiridis I.G., Martin A., Morlon B., Martin L., Cometti G., Pousson M., van Hoecke J. (1996). Co-activation and tension-regulating phenomena during isokinetic knee extension in sedentary and highly skilled humans. Eur. J. Appl. Physiol..

[24] Hollander D.B., Kraemer R.R., Kilpatrick M.W., Ramadan Z.G., Reeves G.V., Francois M., Hebert E.P., Tryniecki J.L. (2007). Maximal eccentric and concentric strength discrepancies between young men and women for dynamic resistance exercise. J. Strength Cond. Res..

[25] Hortobagyi T., Katch F. (1990). Eccentric and concentric torque velocity relationships during arm flexion and extension: Influence of strength level. Eur. J. Appl. Physiol..

[26] Dufour S.P., Lampert E., Doutreleau S., Lonsdorfer-Wolf E., Billat V.L., Piquard F., Richard R. (2004). Eccentric cycle exercise: Training application of specific circulatory adjustments. Med. Sci. Sports Exerc..

[27] Baroni B.M., Stocchero C.M.A., do Espírito Santo R.C., Ritzel C.H., Vaz M.A. (2011). The effect of contraction type on muscle strength, work and fatigue in maximal isokinetic exercise. Isokinet Exerc. Sci..

[28] Hyldahl R.D., Olson T., Welling T., Groscost L., Parcell A.C. (2014). Satellite cell activity is differentially affected by contraction mode in human muscle following a work-matched bout of exercise. Front. Physiol..

[29] Moore D.R., Phillips S.M., Babraj J.A., Smith K., Rennie M.J. (2005). Myofibrillar and collagen protein synthesis in human skeletal muscle in young men after maximal shortening and lengthening contractions. Am. J. Physiol. Endocrinol. Metab..

[30] Eliasson J., Elfegoun T., Nilsson J., Kohnke R., Ekblom B., Blomstrand E. (2006). Maximal lengthening contractions increase p70 s6 kinase phosphorylation in human skeletal muscle in the absence of nutritional supply. Am. J. Physiol. Endocrinol. Metab..

[31] Proske U., Morgan D.L. (2001). Muscle damage from eccentric exercise: Mechanism, mechanical signs, adaptation and clinical adaptations. J. Physiol..

[32] Chapman D., Newton M., Sacco P., Nosaka K. (2006). Greater muscle damage induced by fast versus slow velocity eccentric exercise. Int. J. Sports Med..

[33] Schoenfeld B.J. (2012). Does exercise-induced muscle damage play a role in skeletal muscle hypertrophy?. J. Strength Cond. Res..

[34] Cheung K., Hume P.A., Maxwell L. (2003). Delayed onset muscle soreness: Treatment strategies and performance factors. Sports Med..

[35] Elmer S.J., Martin J.C. (2010). Joint-specific power loss after eccentric exercise. Med. Sci. Sports Exerc..

[36] Paschalis V., Giakas G., Baltzopoulos V., Jamurtas A.Z., Theoharis V., Kotzamanidis C., Koutedakis Y. (2007). The effects of muscle damage following eccentric exercise on gait biomechanics. Gait Posture.

[37] McHugh M.P. (2003). Recent advances in the understanding of the repeated bout effect: The protective effect against muscle damage from a single bout of eccentric exercise. Scand J. Med. Sci. Sports.

[38] Chen T.C., Yang T.J., Huang M.J., Wang H.S., Tseng K.W., Chen H.L., Nosaka K. (2019). Damage and the repeated bout effect of arm, leg, and trunk muscles induced by eccentric resistance exercises. Scand. J. Med. Sci. Sports.

[39] Cermak N.M., Snijders T., McKay B.R., Parise G., Verdijk L.B., Tarnopolsky M.A., Gibala M.J., Van Loon L.J.C. (2013). Eccentric exercise increases satellite cell content in type ii muscle fibers. Med. Sci. Sports Exerc..

[40] Tannerstedt J., Apró W., Blomstrand E. (2009). Maximal lengthening contractions induce different signalling responses in the type i and type ii fibers of human skeletal muscle. J. Appl. Physiol. (1985).

[41] Vijayan K., Thompson J.L., Norenberg K.M., Fitts R.H., Riley D.A. (2001). Fiber-type susceptibility to eccentric contraction-induced damage of hindlimb-unloaded rat al muscles. J. Physiol..

[42] Roig M., O’Brien K., Kirk G., Murray R., McKinnon P., Shadgan B., Reid W.D. (2009). The effects of eccentric versus concentric resistance training on muscle strength and mass in healthy adults: A systematic review with meta-analysis. Br. J. Sports Med..

[43] Higbie E.J., Cureton K.J., Warren G.L., Prior B.M. (1996). Effects of concentric and eccentric training on muscle strength, cross-sectional area, and neural activation. J. Appl. Physiol. (1985).

[44] Aagaard P. (2003). Training-induced changes in neural function. Exerc. Sport Sci. Rev..

[45] Farthing J.P., Chilibeck P.D. (2003). The effects of eccentric and concentric training at different velocities on muscle hypertrophy. Eur. J. Appl. Physiol..

[46] Paddon-Jones D., Leveritt M., Lonergan A., Abernethy P. (2001). Adaptation to chronic eccentric exercise in humans: The influence of contraction velocity. Eur. J. Appl. Physiol..

[47] Douglas J., Pearson S., Ross A., McGuigan M. (2018). Effects of accentuated eccentric loading on muscle properties, strength, power and speed in resistance-trained rugby players. J. Strength Cond. Res..

[48] Leong C.H., McDermott W.J., Elmer S.J., Martin J.C. (2014). Chronic eccentric cycling improves quadriceps muscle structure and maximum cycling power. Int. J. Sports Med..

[49] Cormie P., McGuigan M.R., Newton R.U. (2011). Developing maximal neuromuscular power: Part 1—Biological basis of maximal power production. Sports Med..

[50] Maffiuletti N.A., Aagaard P., Blazevich A.J., Folland J.P., Tillin N., Duchateau J. (2016). Rate of force development: Physiological and methodological considerations. Eur. J. Appl. Physiol..

[51] Franchi M.V., Atherton P.J., Reeves N.D., Fluck M., Williams J., Mitchell W.K., Selby A., Beltran Valls R.M., Narici M.V. (2014). Architectural, functional and molecular responses to concentric and eccentric loading in human skeletal muscle. Acta Physiol..

[52] Timmins R.G., Ruddy J.D., Presland J., Maniar N., Shield A.J., Williams M.D., Opar D.A. (2016). Architectural changes of the biceps femoris long head after concentric or eccentric training. Med. Sci. Sports Exerc..

[53] Seynnes O.R., de Boer M., Narici M.V. (2007). Early skeletal muscle hypertrophy and architectural changes in response to high-intensity resistance training. J. Appl. Physiol. (1985).

[54] Sacks R.D., Roy R.R. (1982). Architecture of the hind limb muscles of cats: Functional significance. J. Morphol..

[55] Friedmann-Bette B., Bauer T., Kinscherf R., Vorwald S., Klute K., Bischoff D., Müller H., Weber M.A., Metz J., Kauczor H.U. (2010). Effects of strength training with eccentric overload on muscle adaptation in male athletes. Eur. J. Appl. Physiol..

[56] Vikne H., Refsnes P.E., Ekmark M., Medbø J.I., Gundersen V., Gundersen K. (2006). Muscular performance after concentric and eccentric exercise in trained men. Med. Sci. Sports Exerc..

[57] Schiaffino S., Reggiani C. (2010). Fibre types in mammalian skeletal muscle. Physiol. Rev..

[58] Ruegg J.C. (1987). Excitation-contraction coupling in fast- and slow-twitch muscle fibers. Int. J. Sports Med..

[59] Farup J., Rahbek S.K., Vendelbo M.H., Matzon A., Hindhede J., Bejder A., Ringaard S., Vissing K. (2014). Whey protein hydrolysate augments tendon and muscle hypertrophy independent of resistance exercise contraction mode. Scand. J. Med. Sci. Sports.

[60] Malliaras P., Kamal B., Nowell A., Farley T., Dhamu H., Simpson V., Morrissey D., Langberg H., Maffulli N., Reeves N.D. (2013). Patellar tendon adaptation in relation to load-intensity and contraction type. J. Biomech..

[61] Coratella G., Schena F. (2016). Eccentric resistance training increases and retains maximal strength, muscle endurance, and hypertrophy in trained men. Appl. Physiol. Nutr. Metab..

[62] Moolyk A.N., Carey J.P., Chiu L.Z.F. (2013). Characteristics of lower extremity work during the impact phase of jumping and weightlifting. J. Strength Cond. Res..

[63] Suchomel T.J., Lake J.P., Comfort P. (2017). Load absorption force-time characteristics following the second pull of weightlifting derivatives. J. Strength Cond. Res..

[64] Comfort P., Williams R., Suchomel T.J., Lake J.P. (2017). A comparison of catch phase force-time characteristics during clean derivatives from the knee. J. Strength Cond. Res..

[65] Suchomel T.J., Taber C.B., Wright G.A. (2016). Jump shrug height and landing forces across various loads. Int. J. Sports Physiol. Perform..

[66] Lake J.P., Mundy P.D., Comfort P., McMahon J.J., Suchomel T.J., Carden P. (2018). The effect of barbell load on vertical jump landing force-time characteristics. J. Strength Cond. Res..

[67] Peñailillo L., Blazevich A., Numazawa H., Nosaka K. (2013). Metabolic and muscle damage profiles of concentric versus repeated eccentric cycling. Med. Sci. Sports Exerc..

[68] Clos P., Laroche D., Stapley P.J., Lepers R. (2019). Neuromuscular and perceptual responses to sub-maximal eccentric cycling: A mini-review. Front. Physiol..

[69] Spiteri T., Nimphius S., Hart N.H., Specos C., Sheppard J.M., Newton R.U. (2014). Contribution of strength characteristics to change of direction and agility performance in female basketball athletes. J. Strength Cond. Res..

[70] Bird S.P., Tarpenning K.M., Marino F.E. (2005). Designing resistance training programmes to enhance muscular fitness. Sports Med..

[71] Medicine A.C.O.S. (2009). American college of sports medicine position stand. Progression models in resistance training for healthy adults. Med. Sci. Sports Exerc..

[72] Headley S.A., Henry K., Nindl B.C., Thompson B.A., Kraemer W.J., Jones M.T. (2011). Effects of lifting tempo on one repetition maximum and hormonal responses to a bench press protocol. J. Strength Cond. Res..

[73] Shibata K., Takizawa K., Nosaka K., Mizuno M. (2018). Effects of prolonging eccentric phase duration in parallel back-squat training to momentary failure on muscle cross-sectional area, squat one repetition maximum, and performance tests in university soccer players. J. Strength Cond. Res..

[74] Schoenfeld B.J., Ogborn D.I., Krieger J.W. (2015). Effect of repetition duration during resistance training on muscle hypertrophy: A systematic review and meta-analysis. Sports Med..

[75] Van den Tillaar R. (2019). Effect of descent velocity upon muscle activation and performance in two-legged free weight back squats. Sports.

[76] Burd N.A., Andrews R.J., West D.W., Little J.P., Cochran A.J., Hector A.J., Cashaback J.G., Gibala M.J., Potvin J.R., Baker S.K. (2012). Muscle time under tension during resistance exercise stimulates differential muscle protein sub-fractional synthetic responses in men. J. Physiol..

[77] Pereira P.E.A., Motoyama Y.L., Esteves G.J., Quinelato W.C., Botter L., Tanaka K.H., Azevedo P. (2016). Resistance training with slow speed of movement is better for hypertrophy and muscle strength gains than fast speed of movement. Int. J. Appl. Exerc. Physiol..

[78] Martins-Costa H.C., Diniz R.C.R., Lima F.V., Machado S.C., Almeida R.S.V.d., Andrade A.G.P.d., Chagas M.H. (2016). Longer repetition duration increases muscle activation and blood lactate response in matched resistance training protocols. Motriz: Revista de Educação Física.

[79] Lacerda L.T., Martins-Costa H.C., Diniz R.C., Lima F.V., Andrade A.G., Tourino F.D., Bemben M.G., Chagas M.H. (2016). Variations in repetition duration and repetition numbers influence muscular activation and blood lactate response in protocols equalized by time under tension. J. Strength Cond. Res..

[80] Munn J., Herbert R.D., Hancock M.J., Gandevia S.C. (2005). Resistance training for strength: Effect of number of sets and contraction speed. Med. Sci. Sports Exerc..

[81] Keeler L.K., Finkelstein L.H., Miller W., Fernhall B. (2001). Early-phase adaptations of traditional-speed vs. Superslow resistance training on strength and aerobic capacity in sedentary individuals. J. Strength Cond. Res..

[82] Pryor R.R., Sforzo G.A., King D.L. (2011). Optimizing power output by varying repetition tempo. J. Strength Cond. Res..

[83] Nóbrega S.R., Barroso R., Ugrinowitsch C., da Costa J.L.F., Alvarez I.F., Barcelos C., Libardi C.A. (2018). Self-selected vs. Fixed repetition duration: Effects on number of repetitions and muscle activation in resistance-trained men. J. Strength Cond. Res..

[84] Martino F., Perestrelo A.R., Vinarský V., Pagliari S., Forte G. (2018). Cellular mechanotransduction: From tension to function. Front. Physiol..

[85] Franchi M.V., Longo S., Mallinson J., Quinlan J.I., Taylor T., Greenhaff P.L., Narici M.V. (2018). Muscle thickness correlates to muscle cross-sectional area in the assessment of strength training-induced hypertrophy. Scand. J. Med. Sci. Sports.

[86] Stasinaki A.-N., Zaras N., Methenitis S., Bogdanis G., Terzis G. (2019). Rate of force development and muscle architecture after fast and slow velocity eccentric training. Sports.

[87] Wilk M., Golas A., Stastny P., Nawrocka M., Krzysztofik M., Zajac A. (2018). Does tempo of resistance exercise impact training volume?. J. Hum. Kinet..

[88] Diniz R.C., Martins-Costa H.C., Machado S.C., Lima F.V., Chagas M.H. (2014). Repetition duration influences ratings of perceived exertion. Percept. Motor Skills.

[89] Tran Q.T., Docherty D., Behm D. (2006). The effects of varying time under tension and volume load on acute neuromuscular responses. Eur. J. Appl. Physiol..

[90] Libardi C.A., Nogueira F.R.D., Vechin F.C., Conceição M.S., Bonganha V., Chacon-Mikahil M.P.T. (2013). Acute hormonal responses following different velocities of eccentric exercise. Clin. Physiol. Funct. Imaging.

[91] Berg H.E., Tesch A. (1994). A gravity-independent ergometer to be used for resistance training in space. Aviat. Space Environ. Med..

[92] Tesch P.A., Ekberg A., Lindquist D.M., Trieschmann J.T. (2004). Muscle hypertrophy following 5-week resistance training using a non-gravity-dependent exercise system. Acta Physiol. Scand..

[93] Norrbrand L., Fluckey J.D., Pozzo M., Tesch P.A. (2008). Resistance training using eccentric overload induces early adaptations in skeletal muscle size. Eur. J. Appl. Physiol..

[94] Fernandez-Gonzalo R., Lundberg T.R., Alvarez-Alvarez L., de Paz J.A. (2014). Muscle damage responses and adaptations to eccentric-overload resistance exercise in men and women. Eur. J. Appl. Physiol..

[95] Maroto-Izquierdo S., García-López D., de Paz J.A. (2017). Functional and muscle-size effects of flywheel resistance training with eccentric-overload in professional handball players. J. Hum. Kinet..

[96] Romero-Rodriguez D., Gual G., Tesch P.A. (2011). Efficacy of an inertial resistance training paradigm in the treatment of patellar tendinopathy in athletes: A case-series study. Phys. Ther. Sport.

[97] Naczk M., Naczk A., Brzenczek-Owczarzak W., Arlet J., Adach Z. (2016). Impact of inertial training on strength and power performance in young active men. J. Strength Cond. Res..

[98] De Hoyo M., Pozzo M., Sañudo B., Carrasco L., Gonzalo-Skok O., Domínguez-Cobo S., Morán-Camacho E. (2015). Effects of a 10-week in-season eccentric-overload training program on muscle-injury prevention and performance in junior elite soccer players. Int. J. Sports Physiol. Perform..

[99] Askling C., Karlsson J., Thorstensson A. (2003). Hamstring injury occurrence in elite soccer players after preseason strength training with eccentric overload. Scand. J. Med. Sci. Sports.

[100] Norrbrand L., Tous-Fajardo J., Vargas R., Tesch P.A. (2011). Quadriceps muscle use in the flywheel and barbell squat. Aviat. Space Environ. Med..

[101] Pozzo M., Alkner B., Norrbrand L., Farina D., Tesch P.A. (2006). Muscle-fiber conduction velocity during concentric and eccentric actions on a flywheel exercise device. Muscle Nerve.

[102] Gonzalo-Skok O., Tous-Fajardo J., Valero-Campo C., Berzosa C., Bataller A.V., Arjol-Serrano J.L., Moras G., Mendez-Villanueva A. (2017). Eccentric overload training in team-sports functional performance: Constant bilateral vertical vs. Variable unilateral multidirectional movements. Int. J. Sports Physiol. Perform..

[103] Maroto-Izquierdo S., García-López D., Fernandez-Gonzalo R., Moreira O.C., González-Gallego J., de Paz J.A. (2017). Skeletal muscle functional and structural adaptations after eccentric overload flywheel resistance training: A systematic review and meta-analysis. J. Sci. Med. Sport.

[104] Sanchez F.J.N., de Villarreal E.S.S. (2017). Does flywheel paradigm training improve muscle volume and force? A meta-analysis. J. Strength Cond. Res..

[105] Vicens-Bordas J., Esteve E., Fort-Vanmeerhaeghe A., Bandholm T., Thorborg K. (2018). Is inertial flywheel resistance training superior to gravity-dependent resistance training in improving muscle strength? A systematic review with meta-analyses. J. Sci. Med. Sport.

[106] Vicens-Bordas J., Esteve E., Fort-Vanmeerhaeghe A., Bandholm T., Thorborg K. (2018). Letter to the editor: Skeletal muscle functional and structural adaptations after eccentric overload flywheel resistance training: A systematic review and meta-analysis. J. Sci. Med. Sport.

[107] Carroll K.M., Wagle J.P., Sato K., Taber C.B., Yoshida N., Bingham G.E., Stone M.H. (2018). Characterising overload in inertial flywheel devices for use in exercise training. Sports Biomech..

[108] Berg H.E., Tesch P.A. (1998). Force and power characteristics of a resistive exercise device for use in space. Acta Astronautica.

[109] Martinez-Aranda L.M., Fernandez-Gonzalo R. (2017). Effects of inertial setting on power, force, work, and eccentric overload during flywheel resistance exercise in women and men. J. Strength Cond. Res..

[110] Lundberg T.R., García-Gutiérrez M.T., Mandic M., Lilja M., Fernandez-Gonzalo R. (2019). Regional and muscle-specific adaptations in knee extensor hypertrophy using flywheel vs. Conventional weight-stack resistance exercise. Appl. Physiol. Nutr. Metab..

[111] Tøien T., Haglo H.P., Unhjem R.J., Hoff J., Wang E. (2018). Maximal strength training: The impact of eccentric overload. J. Neurophysiol..

[112] Sabido R., Hernández-Davó J.L., Pereyra-Gerber G.T. (2017). Influence of different inertial loads on basic training variables during the flywheel squat exercise. Int. J. Sports Physiol. Perform..

[113] Norrbrand L., Pozzo M., Tesch P.A. (2010). Flywheel resistance training calls for greater eccentric muscle activation than weight training. Eur. J. Appl. Physiol..

[114] De Hoyo M., Sañudo B., Carrasco L., Domínguez-Cobo S., Mateo-Cortes J., Cadenas-Sánchez M.M., Nimphius S. (2015). Effects of traditional versus horizontal inertial flywheel power training on common sport-related tasks. J. Hum. Kinet..

[115] Piqueras-Sanchiz F., Martin-Rodriguez S., Martinez-Aranda L.M., Lopes T.R., Raya-Gonzalez J., Garcia-Garcia O., Nakamura N.Y. (2019). Effects of moderate vs. High iso-inertial loads on power, velocity, work and hamstring contractile function after flywheel resistance exercise. PLoS ONE.

[116] Wagle J.P., Taber C.B., Cunanan A.J., Bingham G.E., Carroll K.M., DeWeese B.H., Sato K., Stone M.H. (2017). Accentuated eccentric loading for training and performance: A review. Sports Med..

[117] Aboodarda S.J., Byrne J.M., Samson M., Wilson B.D., Mokhtar A.H., Behm D.G. (2014). Does performing drop jumps with additional eccentric loading improve jump performance?. J. Strength Cond. Res..

[118] Aboodarda S.J., Yusof A., Osman N.A.A., Thompson M.W., Mokhtar A.H. (2013). Enhanced performance with elastic resistance during the eccentric phase of a countermovement jump. Int. J. Sports Physiol. Perform..

[119] Bridgeman L.A., Gill N.D., Dulson D.K., McGuigan M.R. (2016). The effect of exercise induced muscle damage after a bout of accentuated eccentric load drop jumps and the repeated bout effect. J. Strength Cond. Res..

[120] Bridgeman L.A., McGuigan M.R., Gill N.D., Dulson D. (2016). The effects of accentuated eccentric loading on the drop jump exercise and the subsequent postactivation potentiation response. J. Strength Cond. Res..

[121] Hughes J.D., Massiah R.G., Clarke R.D. (2016). The potentiating effect of an accentuated eccentric load on countermovement jump performance. J. Strength Cond. Res..

[122] Sheppard J., Hobson S., Barker M., Taylor K., Chapman D., McGuigan M., Newton R. (2008). The effect of training with accentuated eccentric load counter-movement jumps on strength and power characteristics of high-performance volleyball players. Int. J. Sports Sci. Coach..

[123] Sheppard J., Newton R., McGuigan M. (2007). The effect of accentuated eccentric load on jump kinetics in high-performance volleyball players. Int. J. Sports Sci. Coach..

[124] Sheppard J.M., Young K. (2010). Using additional eccentric loads to increase concentric performance in the bench throw. J. Strength Cond. Res..

[125] Gehlert S., Suhr F., Gutsche K., Willkomm L., Kern J., Jacko D., Knicker A., Schiffer T., Wackerhage H., Bloch W. (2015). High force development augments skeletal muscle signalling in resistance exercise modes equalized for time under tension. Pflügers Archiv Eur. J. Physiol..

[126] Wagle J., Taber C., Carroll K., Cunanan A., Sams M., Wetmore A., Bingham G., DeWeese B., Sato K., Stuart C. (2018). Repetition-to-repetition differences using cluster and accentuated eccentric loading in the back squat. Sports.

[127] Wagle J.P., Cunanan A.J., Carroll K.M., Sams M.L., Wetmore A., Bingham G.E., Taber C.B., DeWeese B.H., Sato K., Stuart C.A. (2018). Accentuated eccentric loading and cluster set configurations in the back squat: A kinetic and kinematic analysis. J. Strength Cond. Res..

[128] Brandenburg J.P., Docherty D. (2002). The effects of accentuated eccentric loading on strength, muscle hypertrophy, and neural adaptations in trained individuals. J. Strength Cond. Res..

[129] Friedmann B., Kinscherf R., Vorwald S., Muller H., Kucera K., Borisch S., Richter G., Bartsch P., Billeter R. (2004). Muscular adaptations to computer-guided strength training with eccentric overload. Acta Physiol. Scand. J..

[130] Yarrow J.F., Borsa P.A., Borst S.E., Sitren H.S., Stevens B.R., White L.J. (2008). Early-phase neuroendocrine responses and strength adaptations following eccentric-enhanced resistance training. J. Strength Cond. Res..

[131] Komi P.V. (1984). Physiological and biomechanical correlates of muscle function: Effects of muscle structure and stretch-shortening cycle on force and speed. Exerc. Sport Sci. Rev..

[132] Sweeney H., Bowman B.F., Stull J.T. (1993). Myosin light chain phosphorylation in vertebrate striated muscle: Regulation and function. Am. J. Physiol. Cell Physiol..

[133] Rassier D., Macintosh B. (2000). Coexistence of potentiation and fatigue in skeletal muscle. Braz. J. Med. Biol. Res..

[134] Sale D. (2004). Postactivation potentiation: Role in performance. Br. J. Sports Med..

[135] Sale D.G. (2002). Postactivation potentiation: Role in human performance. Exerc. Sport Sci. Rev..

[136] Doan B.K., Newton R.U., Marsit J.L., Triplett-McBride N.T., Koziris L.P., Fry A.C., Kraemer W.J. (2002). Effects of increased eccentric loading on bench press 1rm. J. Strength Cond. Res..

[137] Ojasto T., Häkkinen K. (2009). Effects of different accentuated eccentric load levels in eccentric-concentric actions on acute neuromuscular, maximal force, and power responses. J. Strength Cond. Res..

[138] Cormie P., McGUIGAN M.R., Newton R.U. (2010). Changes in the eccentric phase contribute to improved stretch-shorten cycle performance after training. Med. Sci. Sports Exerc..

[139] Griffiths R. (1991). Shortening of muscle fibres during stretch of the active cat medial gastrocnemius muscle: The role of tendon compliance. J. Physiol..

[140] Moore C.A., Weiss L.W., Schilling B.K., Fry A.C., Li Y. (2007). Acute effects of augmented eccentric loading on jump squat performance. J. Strength Cond. Res..

[141] Thys H., Faraggiana T., Margaria R. (1972). Utilization of muscle elasticity in exercise. J. Appl. Physiol..

[142] Wilt F. (1978). Plyometrics: What it is and how it works. Modern athlete and coach. Mod. Athl. Coach..

[143] Asmussen E., Bonde-Petersen F. (1974). Storage of elastic energy in skeletal muscles in man. Acta Physiol. Scand..

[144] Cavagna G.A., Saibene F.P., Margaria R. (1965). Effect of negative work on the amount of positive work performed by an isolated muscle. J. Appl. Physiol..

[145] Myer G.D., Ford K.R., Brent J.L., Hewett T.E. (2006). The effects of plyometric vs. Dynamic stabilization and balance training on power, balance, and landing force in female athletes. J. Strength Cond. Res..

[146] Singh J., Appleby B., Lavender A. (2018). Effect of plyometric training on speed and change of direction ability in elite field hockey players. Sports.

[147] Verkhoshansky Y. (1986). Speed-strength preparation and development of strength endurance of athletes in various specializations. Soviet Sports Rev..

[148] Ebben W.P., Fauth M.L., Kaufmann C.E., Petushek E.J. (2010). Magnitude and rate of mechanical loading of a variety of exercise modes. J. Strength Cond. Res..

[149] De Villarreal E.S.S., Kellis E., Kraemer W.J., Izquierdo M. (2009). Determining variables of plyometric training for improving vertical jump height performance: A meta-analysis. J. Strength Cond. Res..

[150] Markovic G. (2007). Does plyometric training improve vertical jump height? A meta-analytical review. Br. J. Sports Med..

[151] Stojanović E., Ristić V., McMaster D.T., Milanović Z. (2017). Effect of plyometric training on vertical jump performance in female athletes: A systematic review and meta-analysis. Sports Med..

[152] Moran J., Clark C.C.T., Ramirez-Campillo R., Davies M.J., Drury B. (2018). A meta-analysis of plyometric training in female youth: Its efficacy and shortcomings in the literature. J. Strength Cond. Res..

[153] Johnson B.A., Salzberg C.L., Stevenson D.A. (2011). A systematic review: Plyometric training programs for young children. J. Strength Cond. Res..

[154] De Villarreal E.S.S., Requena B., Cronin J.B. (2012). The effects of plyometric training on sprint performance: A meta-analysis. J. Strength Cond. Res..

[155] Asadi A., Arazi H., Young W.B., de Villarreal E.S.S. (2016). The effects of plyometric training on change-of-direction ability: A meta-analysis. Int. J. Sports Physiol. Perform..

[156] Asadi A., Arazi H., Ramirez-Campillo R., Moran J., Izquierdo M. (2017). Influence of maturation stage on agility performance gains after plyometric training: A systematic review and meta-analysis. J. Strength Cond. Res..

[157] De Villarreal E.S.S., Requena B., Newton R.U. (2010). Does plyometric training improve strength performance? A meta-analysis. J. Sci. Med. Sport.

[158] Rössler R., Donath L., Verhagen E., Junge A., Schweizer T., Faude O. (2014). Exercise-based injury prevention in child and adolescent sport: A systematic review and meta-analysis. Sports Med..

[159] Carter A.B., Kaminski T.W., Douex A.T., Knight C.A., Richards J.G. (2007). Effects of high volume upper extremity plyometric training on throwing velocity and functional strength ratios of the shoulder rotators in collegiate baseball players. J. Strength Cond. Res..

[160] Gelen E., Dede M., Bergun Meric Bingul C.B., Aydin M. (2012). Acute effects of static stretching, dynamic exercises, and high volume upper extremity plyometric activity on tennis serve performance. J. Sports Sci. Med..

[161] Vossen J.F., Kramer J.E., Burke D.G., Vossen D.P. (2000). Comparison of dynamic push-up training and plyometric push-up training on upper-body power and strength. J. Strength Cond. Res..

[162] Ebben W.P., Fauth M.L., Garceau L.R., Petushek E.J. (2011). Kinetic quantification of plyometric exercise intensity. J. Strength Cond. Res..

[163] Ebben W.P., Vanderzanden T., Wurm B.J., Petushek E.J. (2010). Evaluating plyometric exercises using time to stabilization. J. Strength Cond. Res..

[164] Ebben W.P., Simenz C., Jensen R.L. (2008). Evaluation of plyometric intensity using electromyography. J. Strength Cond. Res..

[165] Jensen R.L., Ebben W.P. (2007). Quantifying plyometric intensity via rate of force development, knee joint, and ground reaction forces. J. Strength Cond. Res..

[166] Andrade D.C., Manzo O., Beltrán A.R., Alvares C., Del Rio R., Toledo C., Moran J., Ramirez-Campillo R. (2017). Kinematic and neuromuscular measures of intensity during plyometric jumps. J. Strength Cond. Res..

[167] Van Lieshout K.G., Anderson J.G., Shelburne K.B., Davidson B.S. (2014). Intensity rankings of plyometric exercises using joint power absorption. Clin. Biomech..

[168] Jarvis M.M., Graham-Smith P., Comfort P. (2016). A methodological approach to quantifying plyometric intensity. J. Strength Cond. Res..

[169] Ebben W.P., Suchomel T.J., Garceau L.R., Sato K., Sands W.A., Mizuguchi S. The effect of plyometric training volume on jumping performance. Proceedings of the XXXIInd International Conference of Biomechanics in Sports.

[170] Ebben W.P., Feldmann C.R., Vanderzanden T.L., Fauth M.L., Petushek E.J. (2010). Periodized plyometric training is effective for women, and performance is not influenced by the length of post-training recovery. J. Strength Cond. Res..

[171] James L.P., Haff G.G., Kelly V.G., Connick M., Hoffman B., Beckman E.M. (2018). The impact of strength level on adaptations to combined weightlifting, plyometric and ballistic training. Scand. J. Med. Sci. Sports.

[172] Cormie P., McGuigan M.R., Newton R.U. (2010). Influence of strength on magnitude and mechanisms of adaptation to power training. Med. Sci. Sports Exerc..

[173] Hackett D., Davies T., Soomro N., Halaki M. (2016). Olympic weightlifting training improves vertical jump height in sportspeople: A systematic review with meta-analysis. Br. J. Sports Med..

[174] Tricoli V., Lamas L., Carnevale R., Ugrinowitsch C. (2005). Short-term effects on lower-body functional power development: Weightlifting vs. Vertical jump training programs. J. Strength Cond. Res..

[175] Teo S.Y., Newton M.J., Newton R.U., Dempsey A.R., Fairchild T.J. (2016). Comparing the effectiveness of a short-term vertical jump versus weightlifting program on athletic power development. J. Strength Cond. Res..

[176] Fatouros I.G., Jamurtas A.Z., Leontsini D., Taxildaris K., Aggelousis N., Kostopoulos N., Buckenmeyer P. (2000). Evaluation of plyometric exercise training, weight training, and their combination on vertical jumping performance and leg strength. J. Strength Cond. Res..

[177] Lyttle A.D., Wilson G.J., Ostrowski K.J. (1996). Enhancing performance, maximal power versus combined weights and plyometrics training. J. Strength Cond. Res..

[178] Adams K., O’Shea J.P., O’Shea K.L., Climstein M. (1992). The effect of six weeks of squat, plyometric and squat-plyometric training on power production. J. Appl. Sport Sci. Res..

[179] Arabatzi F., Kellis E., De Villarreal E.S.S. (2010). Vertical jump biomechanics after plyometric, weight lifting, and combined (weight lifting+ plyometric) training. J. Strength Cond. Res..

[180] De Villarreal E.S.S., Requena B., Izquierdo M., Gonzalez-Badillo J.J. (2013). Enhancing sprint and strength performance: Combined versus maximal power, traditional heavy-resistance and plyometric training. J. Sci. Med. Sport.

[181] De Villarreal E.S.S., Izquierdo M., Gonzalez-Badillo J.J. (2011). Enhancing jump performance after combined vs. Maximal power, heavy-resistance, and plyometric training alone. J. Strength Cond. Res..

